# Two Cases of General Paralysis Successfully Treated by Urotropin

**Published:** 1905-04

**Authors:** N. F. MacHardy

**Affiliations:** Assistant Medical Officer at the Coton Hill Hospital for the Insane, Stafford, England


### Two Cases of General Paralysis Successfully Treated by Urotropin.

By N. F. MacHARDY, M. B., Ch.B.,
Assistant Medical Officer at the Coton Hill Hospital for the Insane, Stafford, England.

[*British Medical Journal*, January 28, 1905.]

The following cases are of interest as showing recoveries,—at least temporary and probably permanent,—from general paralysis of the insane, associated in the first case with locomotor ataxia. The treatment was urotropin. These are the only two cases treated, and both have maintained their recovery up to date of publication:

Case I.—G. W., aged 44; admitted October 11, 1902; dangerous and violent; marked general paralysis. Suffered from locomotor ataxia for over two years before admission, and for one week from mental symptoms. Grandiose delusions as regards wealth and strength. Progress till put on urotropin; delusions became worse. November 13, said he was worth £300,000,000. Steady progression of disease affecting walk and speech till September, 1903, when he was confined to bed, lead a catheter life, and had to be attended to like an infant. By November 15, he became very weak, and I then commenced with 5 gr. urotropin daily.

Progress while on urotropin: December 29, urine is beginning to pass naturally. Patient able to sit up, and be assisted about by an attendant; is talking, but full of delusions.

January 20, 1904, physically much stronger, is up and has regained power over limbs. Passes all urine naturally, is full of delusions, says he is a girl, is a king, is very wealthy, and the like. February 20, considerable mental and physical improvement. Plays billiards, but not well; ataxia diminishing; memory affected and illusional; now on 10 gr. urotropin daily. March 20, talks more distinctly; is losing delusions; plays billiards better; worth only £30,000. April 20, had no lightning pains for three months; plays whist and billiards constantly; lost chief delusions; now on 15 gr. urotropin daily. May 11, memory still imperfect; is still a little boastful, but has no delusions; can talk well; physically very fit. June 10, no lightning pains since January; memory improving; no delusions or signs of general paralysis; pupil contracts to light. July 1, home on probation. August 1, I hear he is still better and now only appears nervous. Is still on 15 gr. urotropin; discharged today. October 1, he is as well as he has been and shows no signs of breaking down.

Case II.—A. F. G., aged 38; admitted May 28, 1904. Attacks since January, 1904. Improvement in February and then became much worse. On admission he had Argyll Robertson pupils and very exaggerated knee-jerks, with ankle clonus, twitching of facial muscles, slurring speech, and the like.

June 2, leads a catheter life. Put on urotropin, 2½ gr. twice daily. Is recovering from his semicataleptic condition, but hardly talks. June 9, some improvement. Passes a little urine by himself and helps to dress himself. Once or twice quite talkative. Told me he was buying a yacht, 250 feet long for £10,000. June 14, improvement continues. Gave him 5 gr. urotropin twice daily, but he had some hematuria, so I stopped it for a time. June 23, has gone back. Catheter life again; 2½ gr. urotropin twice daily. June 29, improving daily. Delusions of exaltation passing. Walks without assistance. Says he is a poor man. Passes water freely. July 8, improved very rapidly. Walks about and talks like a sane man. No delusions. Progress almost unbelievable. July 12, apparently well in every respect. Slight inequality of pupils only physical symptom. July 15, went home on probation. August 16, discharged "cured." September 10, got a letter from him today, very sensible. His friends think he is better than for years. Starting in business again. October 2, still well and continuing urotropin.

In conclusion, I would state that the influence of urotropin is marked from day to day. If the patient shows signs of relapsing, an increased dose stops the tendency. As before remarked, these two cases are the only ones treated, so this method deserves a trial in similar cases, as so far I have no failures to chronicle.

